# Hemorrhage after Extraction

**Published:** 1892-09

**Authors:** A. A. Burns

**Affiliations:** Smith's Falls, Ont.


ARTICLE II.

### HEMORRHAGE AFTER EXTRACTION.

BY A. A. BURNS, L. D. S., SMITH'S FALLS, ONT.

Read before the Eastern Ontario Dental Association.

In this paper I do not purpose dealing with any minute physiological state of the blood or tissues, but more especially to recall to the mind the ready and effective methods of arresting an undue flow of the blood after the extraction of a tooth. It has been stated that the appearance of a patient will be a key as to whether there is a likelihood of any hemorrhagic trouble. After taking due notice of the above, very little warning is given the surgeon from this source.

About the only correct warning is when the patient informs the surgeon of a trouble of this nature on a previous occasion. When a case of this kind is presented, it is well to take every precaution when performing operations of a similar kind for any members of the same family, as the trouble is said to be hereditary, and good grounds are presented from past experiences for so believing.

After a tooth has been extracted, the blood comes spontaneously and flows for an unlimited time in a degree such as will not cause any alarm. Usually after five minutes' flowing there is a process of clotting taking place. This stage in the flow of blood is called primary hemorrhage, and is usually all that the surgeon has to deal with, as the blood generally stops flowing as soon as the clot is formed; but sometimes after the clot has formed, even eight or ten hours after, there appears a flow of blood more rapid than the preceding and in a somewhat pulsating manner. This is known as secondary hemorrhage. When this occurs, it is first advisable to remove the clot which has formed in order that a more definite application may be made.

Some surgeons advise the use of a large burr as a first means of arresting the hemorrhage. The burr is passed up the socket and then given a half turn. This carries out, to an extent, the Torsion method of dealing with hemorrhage. But while this may be all sufficient, it is open to doubt, as frequently the blood comes from the underlying portion of gum, and should this be the case, the foregoing method would fail.

What seems more practical is to take a strong solution of alum, formed by dissolving alum in warm water, and first applying with a syringe; after a few applications in this manner, pellets of cotton may be saturated with the solution and forced into the socket. Some think that a greater effect is produced by applying powdered alum to the pellet after saturation. This may make the action more powerful. However, if this not effectual, it is well to repeat the treatment; or should we prefer another treatment to this: the wound may be syringed with peroxide of hydrogen, which is said to have an immediate action causing a clot which is not soluble in the blood. Pellets may also be saturated and inserted into wound.

While the patient is being treated, it is well to lose no time as patient is growing weaker, and the blood is losing its clotting action on account of one of its exponent parts, namely, the serum becoming greater in proportion, displacing the. organic matter. If none of these methods are successful, the stronger styptics must be resorted to.

The following are classed among the more powerful styptics for local application: Nitrate of silver, tannic acid, subsulphate of iron, perchloride of iron, persulphate of iron, gallic acid, tincture of ergot. Care must be taken so that no agent is employed as a styptic which will in any way destroy the tissue.

Nitrate of silver may prove successful in some cases, but it causes destruction of the tissues with which it comes in contact, and also forms a clot which is soluble in blood. Perchloride of iron acts in much the same manner.

Persulphate of iron is the best of the iron compounds. It acts readily, does not destroy tissue, and after action presents a clean looking wound.

Tannic acid is an excellent styptic, and answers well in connection with a compress of lint or cotton. Also gallic acid. The clot formed by these is not soluble in blood.

Powdered subsulphate of iron used on pellets of cotton saturated with sandarac varnish, followed by the use of the compress so adjusted as to act directly upon the mouth of the bleeding vessel. This is generally effective in alveolar hemorrhage.

Tincture of ergot is also good, but must be used hypodermically in about the proportion of one part ergot to two parts water. It is found that when the tissue has been punctured by the needle it presents a dark, swollen and unsightly appearance. During the time that operation is going on, it is well to see that the patient rests, and is in the horizontal position, having the head and shoulders raised. It is well in severe cases to treat other ways than locally. Arterial sedatives should be administered, such as opium one grain, and acetate of lead two-third grains. Opium should not be administered in this quantity more frequently than once every three hours, and then as few times as possible.

If it is found neces ary to resort to other means, the following might be of use: Take a piece of compound, and having heated it place it upon the jaw directly opposite the wound. Have patient close jaw, and in order that they do not meet closely, it is advisable to place two little blocks of wood in the compound before inserting; when this is properly hardened, it may be removed. The part of compound which came in contact with the wound should now be removed, leaving only a sufficient quantity to serve as a division between the jaws.

Take a quantity of plaster of Paris, and mix it to the proper consistency, using alum water for the purpose. It is necessary to hasten in doing this, as the plaster when mixed in this manner hardens quickly. Fill the cavity recently formed in the compound and place again on the jaw requesting the patient to close as before, and then having bandage ready pass it under the chin, allowing the two ends to meet over the hard, where they are securely fastened. In this manner the jaws are caused to remain in a fixed position.

In any treatment it is well to leave local applications, such as pellets of cotton, compress, etc., in position until there is not the least danger of a récurrence of bleeding. Some even go as far as to allow the pellets to remain in until they are thrown off by nature.

While the object of this paper has been to try and throw out a few ideas for use in any case of severe hemorrhage, if you will permit, I will spend a few minutes in the general treatment after every case of extraction.

I consider it best to syringe out the wound until a sufficient quantity of acid has flowed from wound, and endeavor as far as possible to carry this out. It will be found that if a slow stream of cold water is poured upon the bleeding tissue with the aid of a stringe, the blood will almost immediately cease flowing.

While the treatment may be somewhat heroic in general prractice for winter, it might be more agreeable to patient to dispense with the above. As a substitute take carbolic acid and glycerine in proportion of one to four, and in about a quarter glass of tepid water; drop from six to ten drops. This will be found to be equally as effectual as the other.

Some patients are given to what may be called continuous sucking of the wound immediately after a tooth has been removed. Others keep up a constant spitting. Blood is found to clot more quickly when allowed to flow of its own free will, so that either or both of the above actions are a great hindrance to *nature's* method.—*Dominion Dent. Journal.*

